# "Night-Nurse's Paralysis": A Temporary Tonic Motor Paralysis
*Paper read before Bristol Medico-Chirurgical Society, 8th May, 1946.


**Published:** 1946

**Authors:** G. de M. Rudolf

**Affiliations:** Hon. Psychotherapist, West End Hospital for Nervous Diseases; Psychiatric Specialist, Ministry of Pensions


### "NIGHT-NURSE'S PARALYSIS": A TEMPORARY TONIC MOTOR PARALYSIS.*

BY

G. de M. Rudolf, M.R.C.P., D.P.M., D.P.H.

*Hon. Psychotherapist, West End Hospital for Nervous Diseases; Psychiatric Specialist, Ministry of Pensions.*

---

On a beautiful evening of April, 1943, I gave a talk on elementary psychology to a small party of R.A.M.C. orderlies and convalescent patients at a detached hospital at Gibraltar. But this simple talk led up to a series of events of which to-night is one. At the end of my short talk, an orderly rose and said: "Sir, will you tell us what night-nurse's paralysis is?" I looked at him, thought, looked at him again, tried further thought, and finally said: "No, certainly not. I've never heard of it." The orderly then gave a full description of the condition, which was confirmed by two other orderlies in this party of about a dozen men. I returned to my billet a sadder man. After several years of neurology and over twenty years of psychiatry, to be told of a common condition of which I had never heard by a temporary full private of the R.A.M.C. seemed, to put it mildly, humiliating. But a thought occurred—perhaps the hind-limb of the trick-cyclist was being elongated, especially as I was the first psychiatrist to be on the Rock.

So I discussed the condition with other medical officers, but found, to my surprise, that they too were entirely ignorant of it. One Sunday afternoon, the Registrar and I determined to prove if the condition really occurred. Unexpectedly and rapidly, so that our journey could not be passed from ward to ward, we visited the Sisters on duty. One had not heard of the condition, one had known of it in a friend, and one had suffered from it. The proof was sufficient. I then began a systematic individual enquiry from doctors, nurses, and others, with the result seen in Table 1.

* Paper read before Bristol Medico-Chirurgical Society, 8th May, 1946.

Table 1. GIBRALTAR ENQUIRY.

| Occupation | Enquiries from | Suffered from attacks | Knowledge only of condition |
|---|---|---|---|
| Nurses (M. & F.) .. | 67 | 12 | 15 |
| Naval Officers (Executive) | 15 | 2 | 5 |
| Printers .. .. .. | 21 | 2 | 5 |
| Compositors .. .. | 19 | 0 | 1 |
| Radar Operators .. | 60 | 0 | 0 |
| Doctors .. .. .. | 34 | 0 | 1 |

The enquiry comprised the questioning of each person individually as to whether he knew of the existence of the condition, which I described and later named. Whilst this enquiry was being carried out, a party of Consultants visited the Rock. So I asked one of them if he could tell me something about night-nurse's paralysis. He looked at me with an indulgent, kindly smile, not believing that the condition existed. He crossed the hall of the Bristol Hotel and spoke to an Army Sister who had come off the ship with him. But he had "had it"; so had the Sister, three attacks.

No reference to night-nurse's paralysis could be found in text-books, the editor of the *Nursing Mirror* knew of no article on it in the nursing periodicals, senior officers of the War Office A.M.D. were unaware of the existence of the condition, and the Librarian of the Royal Society of Medicine could find nothing about this state in the literature. The condition consists of a sustained immobility involving the entire voluntary musculature, although full consciousness prevails and the subject can see and hear. Awareness of the inability to move is present. The musculature is rigid, and considerable force is needed to push the sufferer aside. Attacks last from a few seconds to as many minutes, and the onset is insidious, the subject suddenly finding he is unable to move. The attack may begin when the individual is standing or sitting or when moving, as in a nurse who was turning over the pages of a report book when he became "fixed" with his hand holding up a page. Attacks occur in the presence of others and when the subject is alone.

Although no ill effect follows an attack, with the exception of a very brief period of confusion if the subject is kicked or shaken out of the condition, serious results of attacks might occur. In one instance, a minelayer, fully-laden, would have collided with a cruiser if a second officer had not been on the bridge of the cruiser and pushed aside the navigating officer and acted for him. A calico-printer watched twenty yards of calico slowly tear without being able to move to stop the machine. A printer watched his paper burn without being able to attempt to extinguish the fire, a near-by foreman doing so.

The number and frequency of attacks vary. One naval officer in nearly forty years had one attack, in which he allowed an electric motor to burn out, as he could not move to turn it off. Another officer passed through forty to fifty attacks in from four to five years. The condition is widespread. I have records of attacks occurring in the Arctic Ocean, Montreal, Belfast, Edinburgh, Glasgow, Liverpool, Macclesfield, St. Albans, London, Woolwich, France, Gibraltar, Salerno and Cairo.

The hospitals in which nurses of this series have been affected have been teaching (Guy's), mental and military.

The nature of the condition and cause of its onset are not clear. It is easy to exclude many states of sustained immobility (Table 2) but less easy to include the condition under any heading.

Table 2. CAUSES OF GENERALIZED IMMOBILITY.

| With Tonic Muscles. | With Flaccid Muscles. |
|---|---|
| Voluntary concentration. | Sleep, usually. |
| Fear. | Cataplexy. |
| Catalepsy. | Narcolepsy. |
| Vaso-vagal attacks. | Coma. |
| Schizophrenia. | Concussion. |
| Post-encephalitic states. | Shock. |
| Day-dreaming. | Hysteria. |
| Epilepsy. | Epilepsy. |
| Hysteria. | |

? Sleep Paralysis. ?

A year ago I read a paper on this condition before the Association of British Neurologists, where the opinion was expressed by a senior neurologist that the state was cataleptic. At this year's meeting, he was less certain. But catalepsy appears to be the most suitable condition with which to identify the state. Many descriptions of catalepsy confine it to *flexibilitas cerea* in which the limbs remain in a position in which they are placed by an observer. But Michell Clarke, forty-two years ago, gave a description of generalized catalepsy which describes this state. The varying appearance of the condition amongst different classes of persons is of interest. The attacks appear to be more likely to occur amongst persons liable to be stationary. Amongst printers the condition is well-known, amongst compositors it is rare. The absence of attacks in radar operators may be due to the usual short period of duty at the screen of thirty minutes.

I was unable to find evidence of the condition in the R.A.F., although its occurrence in pilots flying on " G " might be expected, as on long-flying anti-submarine patrols, where " George," the automatic pilot, is used during the patrol of either twelve or twenty-four hours' duration. Experienced pilots make mistakes towards the end of these patrols, but these may not be due to the tonic-paralysis now being discussed. Unfortunately, my information in this field is too limited to be of value as, although the Powers-that-Were allowed me to observe the Services at duty, as in destroyers in action, in a submarine submerged, or in aircraft over the African desert, they refused to agree to the suggestion of the R.A.F. that I should go in a Catalina on a long patrol.

To recapitulate, the condition known amongst nurses as night-nurse's paralysis occurs amongst others. It consists of a temporary tonic motor paralysis of insidious onset. Consciousness is clear. Its distribution is widespread, it is well known amongst certain classes of persons, but little known amongst doctors. It bears a strong resemblance to catalepsy as described by Michell Clarke, but whether its cause is psychological, chemical or neurological, or a combination of two or three, is not known.

